# Effects of Phase Morphology on Mechanical Properties: Oriented/Unoriented PP Crystal Combination with Spherical/Microfibrillar PET Phase

**DOI:** 10.3390/polym11020248

**Published:** 2019-02-02

**Authors:** Dashan Mi, Yingxiong Wang, Maja Kuzmanovic, Laurens Delva, Yixin Jiang, Ludwig Cardon, Jie Zhang, Kim Ragaert

**Affiliations:** 1 College of Polymer Science and Engineering, State Key Laboratory of Polymer Materials Engineering, Sichuan University, Chengdu 610065, China; midashan@126.com (D.M.); wang137236180@163.com (Y.W.); jyx514037215@163.com (Y.J.); 2 Centre for Polymer and Material Technologies, Department of Materials, Textiles and Chemical Engineering, Faculty of Engineering and Architecture, Ghent University, Technologiepark 915, 9052 Zwijnaarde, Belgium; Maja.Kuzmanovic@Ugent.be (M.K.); Laurens.Delva@Ugent.be (L.D.); Ludwig.Cardon@Ugent.be (L.C.)

**Keywords:** recycle, microfibrillar composites, shish-kebab

## Abstract

In situ microfibrillation and multiflow vibrate injection molding (MFVIM) technologies were combined to control the phase morphology of blended polypropylene (PP) and poly(ethylene terephthalate) (PET), wherein PP is the majority phase. Four kinds of phase structures were formed using different processing methods. As the PET content changes, the best choice of phase structure also changes. When the PP matrix is unoriented, oriented microfibrillar PET can increase the mechanical properties at an appropriate PET content. However, if the PP matrix is an oriented structure (shish-kebab), only the use of unoriented spherical PET can significantly improve the impact strength. Besides this, the compatibilizer polyolefin grafted maleic anhydride (POE-*g*-MA) can cover the PET in either spherical or microfibrillar shape to form a core–shell structure, which tends to improve both the yield and impact strength. We focused on the influence of all composing aspects—fibrillation of the dispersed PET, PP matrix crystalline morphology, and compatibilized interface—on the mechanical properties of PP/PET blends as well as potential synergies between these components. Overall, we provided a theoretical basis for the mechanical recycling of immiscible blends.

## 1. Introduction

Polypropylene (PP) and polyethene terephthalate (PET) are among the most frequently used semi-crystalline polymers, accounting for nearly 30% of all plastics used in Europe [1]. They are commonly used in consumer goods, car parts, packaging, and textiles [2,3]. In some cases, both polymers are used in applications where they are physically fused together in order to combine some of the important characteristics of each component. Examples of this include industrial carpet, which contains PET yarns physically attached to a PP melt backing [4], and multilayer food packaging, in which PET provides barrier properties and laminated PP provides water resistance as well as sealing ability [4]. Unfortunately, this means that at their end-of-life, the polymers cannot be easily separated back into the composing mono-materials PP and PET. Instead, within the bounds of mechanical recycling, they must be processed as an immiscible blend. Such blends are typically inferior in terms of material properties, and, therefore, it is common to incinerate them for energy recovery rather than to effectively recycle them [5]. It is therefore paramount to the sustainability of these PP/PET products to find upcycling strategies that will allow the secondary materials to achieve sufficient properties to make recycling more attractive than incineration. This manuscript investigates the formation of two processing-based methods that alter the blend’s microstructure as techniques for such upcycling as well as the potential synergy between both approaches.

The mechanical properties of thermoplastic blends can typically be improved in the presence of fibers [6,7,8]. In situ generation of short fibers is a possible way to change the PET phase from spherical particles to fibers and form microfibrillar composites (MFC). The MFC concept is a type of polymer-polymer composite in which a high-melting fibrillar thermoplastic polymer reinforces a lower-melting one [9]. The steps to form MFC have been previously described [10]. In brief, they are as follows: At first blend preparation by melt extrusion, followed by continuous cold drawing and granulating. Then, the fibrillated blend is processed (by either injection molding or compression molding) at such temperatures that the PP matrix can return to a homogenized and isotropized state while the dispersed PET phase retains its fibrillar shape.

The influences of cold drawing ratio, PET concentration, injection temperature, and various compatibilizers on the morphology, crystallization behavior, and rheological behavior of polyolefin/PET MFC have been investigated [10,11,12,13,14,15,16]. MFC are easy to recycle and have been noted to show improved thermal stability and flexural modulus [10,17]. Properly designed PP/PET composites can also have a noticeable increase in impact strength [17,18,19,20]. One major challenge for PP/PET composites is that the composing polymers are inherently incompatible, thus causing a reduction in the final composite strength due to the weak fiber/matrix interaction. In this work, we used polyolefin grafted maleic anhydride (POE-*g*-MA) to reduce the interfacial tension between the PET fiber and the PP matrix. The olefinic segment of POE is compatible with PP, whereas the maleic anhydride can covalently bond with the PET carboxyl groups. In our previous work, we already found that this type of POE-*g*-MA can promote a fine dispersed phase morphology and improve the toughness [21].

Besides the PET, the crystalline morphology of the PP matrix can also greatly influence the properties of PP/PET blends [22,23,24,25]. The distinctive crystallization morphology resulting from a strong flow is often referred to as a shish-kebab. The shish-kebab was discovered in the 1960s and has been extensively studied over the past decades for its ability to dramatically improve polymer properties [26,27,28,29,30,31,32]. The shish-kebab has a huge aspect ratio and can be formed by high shear stress. In that case, the highly oriented shish is formed at first, and then the kebabs can grow on the shish to complete the shish-kebab structure. In this work, multiflow vibrate injection molding (MFVIM) was used to create high shear stress and form a large amount of shish-kebab structure.

It is typical for MFC studies to focus on the fibril morphology and the matrix-fibril-interface as determining factors for the mechanical properties, but the crystalline morphology of the matrix is often disregarded. It is therefore the purpose of this study to investigate the influence of all composing aspects—fibrillation of the dispersed PET, PP matrix crystalline morphology, and compatibilized interface—on the mechanical properties of PP/PET blends as well as potential synergies between these components. In these experiments, PET spherical particles and microfibrils were achieved during the extrusion-based preparation step, while PP spherulite and shish-kebab structures were induced via different injection methods. After investigating the crystal and phases morphologies, this study has identified specific and targeted methods to optimize the properties of PP/PET blends for different PET contents. 

## 2. Experimental Section

### 2.1. Materials and Sample Preparation

#### 2.1.1. Materials

Polypropylene (PP) was purchased from Sabic (Sabic 575P, Bergen op Zoom, the Netherlands) with a melt flow rate (MFR) of 11 g/10 min (2.16 kg, 230 °C). PET (trade name: LIGHTER C93), a bottle-grade material with an intrinsic viscosity of 0.80 ± 0.02 dL/g, was provided by Equipolymers (Schkopau, Germany). POE-*g*-MA was Acti-Tech 16MA13, a Vistamaxx-based compatibilizer donated by the Nordic Grafting Company (NGC, Hellerup, Denmark). The grafting percentage of the MA group onto the backbone of the compatibilizer was 1.3 wt % according to the data sheet. PET was dried in an oven for 12 h at 80 °C and for 2 h before processing at 120 °C.

#### 2.1.2. Sample Preparation

##### Extrusion Methods

Melt blending was achieved by a twin-screw extruder (Coperion ZSK18, Stuttgart, Germany) with two co-rotating screws with a diameter of 18 mm, L/D of 40 and die opening of 19 mm × 2 mm. The screw speed and barrel temperatures were set to 120 rpm and between 205 and 260 °C, respectively. In this study, PP was used as a matrix, different amounts of PET were added, and the content of POE-*g*-MA was maintained at 4 wt %. After passing through calender rolls, the extrudate was obtained as a sheet with a dimension of 25 mm × 1 mm and cooled down to approximately 15 °C. Then, some sheets were shredded before injection molding, and others were drawn in a hot oven (200 °C, 55.5 cm × 60 cm) to form MFC. The measured temperature (IR camera) of the extrudate in the oven was 95 °C. During drawing, the speed of the rolls was adjusted to obtain a draw ratio of 8. More detailed information about the extrusion methods can be found in our previous work [10].

##### Injection Methods

The compound materials were processed by a homemade injection machine after drying in an oven for 2 h at 100 °C. In this experiment, conventional injection molding (CIM) and multiflow vibrate injection molding (MFVIM) were used to fabricate samples. In MFVIM, a mold with a flash groove that can form multiflow in a sample during packing is used. The mold is initially filled with melt at a certain injection pressure; no melt should spill through the flash groove. Oscillatory pressure is then introduced to form second and third flows during packing; at this stage, a portion of the melt could be pushed out of the cavity through the flash groove. Each flow can form a new shear layer with shish-kebabs. The detailed information about the difference between MFVIM and CIM is available in previous research [33,34] and the Figure S1 (support information). The temperature profile values from the hopper to the nozzle were 160, 180, 190, 200, and 200 °C. The mold temperature was set to 40 °C. After injection molding, the samples were cut into certain shapes (dumbbell or strip) along the flow direction for different mechanical tests.

**Figure 1:**
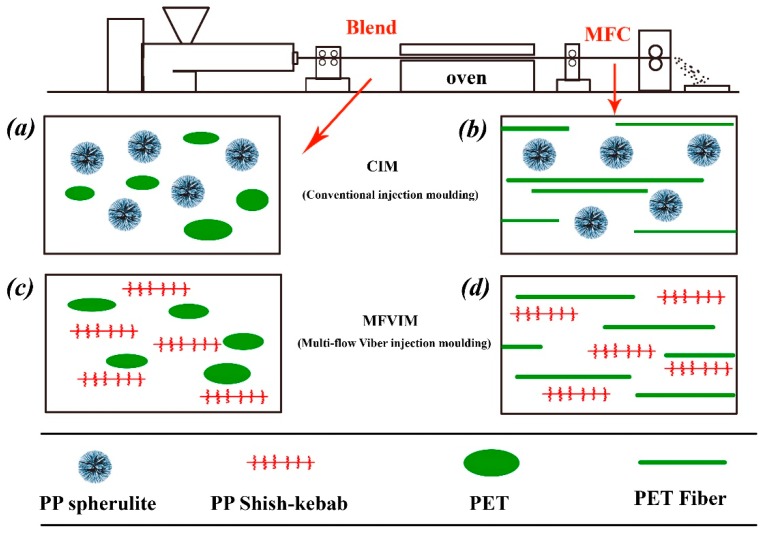
Scheme of experimental setup: (**a**) Blend + conventional injection molding (CIM) [BC], (**b**) microfibrillar composites (MFC) + CIM [MC], (**c**) blend + multiflow vibrate injection molding (MFVIM) [BV] and (**d**) MFC + MFVIM [MV].

The scheme of the experimental setup is shown in Figure 1. Four kinds of phase structures are formed using different processing methods. For each structure, more than eight samples were prepared for various tests. After blending and CIM, the PET phase is dispersed in the PP matrix in a spherical shape, as shown in Figure 1a, whilst the PP phase forms spherical particles due to the low shear stress. As shown in Figure 1b, the PET phase is changed from a spherulite to a microfiber by cold drawing and forms the traditional MFC. When MFVIM is introduced, the shear layer progressively thickens and forms shish-kebabs, as shown in Figure 1c. The high shear stress cannot change the morphology of the PET phase, which still spreads as spherical particles, because the molding temperature is considerably lower than the melting point of PET. In Figure 1d, the MFC and MFVIM are combined. In this way, the PP matrix and PET phase are present in a fibrous form.

The names of the samples are shown in Table 1. For example, BV5 is a sample that contains 5 wt % PET and is molded by MFVIM after simple blending.

### 2.2. Characterization and Testing

#### 2.2.1. Two Dimensional Small Angle X-ray Scattering (2D-SAXS)

Two dimensional small angle X-ray scattering (2D-SAXS) was applied to detect the morphology, using a scatterometer (Xeuss2.0, Xenocs, Sassenage, France). The specimens were 1 mm thick slices cut along the flow direction. The wavelength of light was 0.154 nm, which was created by a Cu tube. The sample-to-detector distance was 2474 mm, and the exposure time was 300 s for each sample.

#### 2.2.2. Scanning Electron Microscopy (SEM)

Morphology was characterized by a JEOL field emission scanning microscopy (model JSM-7500F, Tokyo Japan) with an acceleration voltage of 5 kV. Specimens were cut parallel to the flow direction. Two different solutions were used to etch the amorphous PP or the POE-*g*-MA phase. An acid solution of H_2_SO_4_-H_3_PO_4_-KMNO_4_ was used to etch the amorphous PP and POE-*g*-MA at the same time, as the n-heptane at 50 °C for 7 h will only remove POE-*g*-MA. All the specimens were dried and then coated with a thin layer of gold before SEM characterization. 

#### 2.2.3. Differential Scanning Calorimetry (DSC)

The melting behavior of the PP matrix for the different samples was analyzed by a differential scanning calorimetry (DSC) device (TA Q200) in the temperature range from 40–200 °C with a heating rate of 10 °C /min. The following equation was utilized for calculating the total crystallinity *X_c_* for PP of each sample:

(1) $$X_{c}=\frac{\Delta H_{m}}{\Delta H_{m}^{0}\varphi_{i}}$$

where $\Delta H_{m}$ represents the measured fusion enthalpy, and $\Delta H_{m}^{0}$ is the theoretical fusion enthalpy of completely crystallized PP (207 J/g). $\varphi_{i}$ is the mass fraction of PP in the blend. Then, the samples were cooled to 40 °C with a cooling rate of 10 °C /min for non-isothermal crystallization after isothermal at 200 °C for 10 min. All the DSC measurement were carried out under dry nitrogen atmosphere.

#### 2.2.4. Mechanical Testing

Tensile properties along flow direction were measured by an Instron 5967 machine (Instron Corp., Norwood, MA, USA) with a cross-head speed of 20 mm/min, and the tensile modulus was obtained by line’s slope in the stress-strain curve. Notched Izod impact strength was used to evaluate the toughness of the samples. The impact tests were performed on a XJUD-5.5 Izod machine, and a 2 mm deep V-shaped notch was made for each specimen before the test. At least six measurements were made for each sample and then the average value was reported.

## 3. Results and Discussion

### 3.1. Structure Morphology

**Figure 2:**
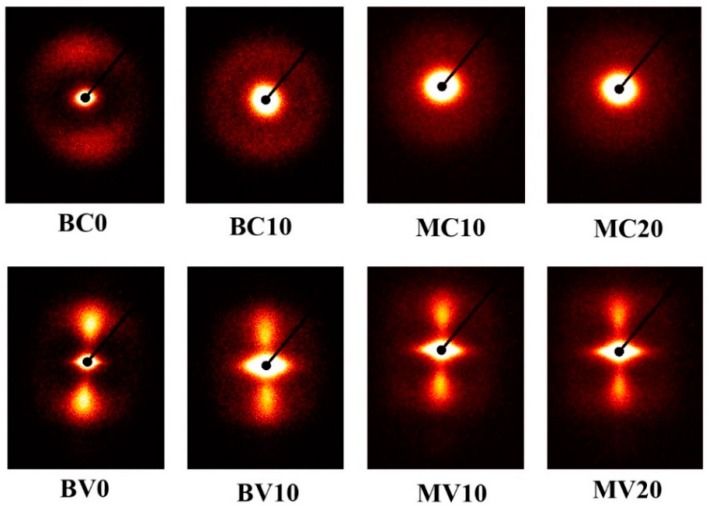
Two dimensional small angle X-ray scattering (2D-SAXS) patterns of some selected samples. The flow direction is perpendicular.

SAXS was used to verify the phase morphologies that is expected in Figure 1. Figure 2 shows a series of SAXS patterns from some selected samples that can illustrate the crystal information of PP/PET blends. Evidently, the sample prepared via CIM showed a typically weak and broad meridional maximum, thereby indicating that almost no oriented lamella existed. For BV0, BV10, MV10, and MV20, a narrow meridional maximum and an equatorial streak were observed. The meridional maxima in the SAXS pattern were due to the formation of some well-oriented lamellae, such as kebabs, which were perpendicular to the flow direction. The equatorial streak was due to the formation of shish or oriented “daughter” lamellae. In conclusion, broad meridional maximum indicates that PP crystals existed as spherulites in the BC and MC. While the equatorial streak and narrow meridional maximum indicate that shish-kebabs existed in the BV and MV samples (Figure 2) [35,36,37]. 

**Figure 3:**
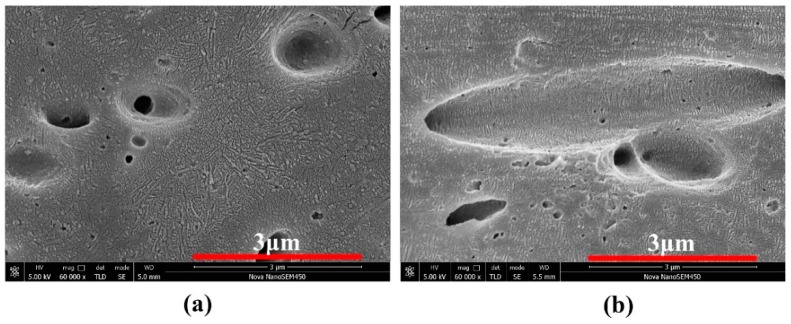
SEM images show the crystal structures of (**a**) BC5, and (**b**) MV5.

Figure 3 shows the morphology of BC5 and MV5, and the viewing surface was parallel to the flow direction. The PET, POE-*g*-MA, and amorphous regions of PP were etched by the acid solution, thereby leaving the PET holes and lamellar PP crystals visible on the matrix. For BC10, no complete spherulites could be observed, and only non-uniform lamellae were present because the added PET can induce crystallization and disturb the crystallization process of PP [38]. Figure 3a also confirms that the PET phase was indeed dispersed in the PP matrix in a spherical shape, and its diameter was less than 3 μm. In our previous work, we found that POE-*g*-MA can greatly decrease the interface tension, thereby resulting in a microscaled dispersed PET phase [21,39]. 

These microscale spherical PET particles with PP shish-kebabs have a synergistic effect on the toughening of PP/PET blends, as discussed later. Only a part of the PET microfibers is observed in Figure 3b because they are not fully oriented along the flow direction, and the other parts of the fiber remain immersed in the matrix. In contrast to the random orientation of PP lamellae in BC10, high oriented lamellae are present in MV5. The oriented lamella should be the kebab lamella that perpendicularly grows toward the shear direction, whereas the shish-kebabs are undetectable because of the overgrown kebabs.

**Figure 4:**
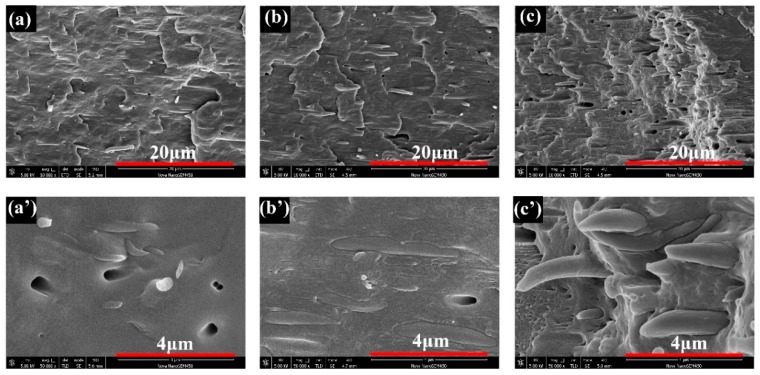
SEM of fracture surface along the flow direction. (**a**)/(**a’**) MV5, (**b**)/(**b’**) MV10, and (**c**)/(**c’**) MV20.

Representative SEM images of the fracture surface along the flow directions of MV5, MV10, and MV20 are shown in Figure 4 to examine the dispersion and distribution of PET. For MV5 (Figure 4a), many microfibers were formed, but their average diameter was particularly small (approximately 0.42 μm). The average diameters of the PET fibers of the MV10 and MV20 samples were approximately 0.66 and 0.70 μm, respectively. Evidently, cold drawing can form particularly long microfibrils. However, these microfibrils are shredded before injection and may break during the injection molding under a high shear rate [10]. The multiflow can also break the thin PET fibers at low PET content, so MV5 is expected to have the shortest fibers. At this point, the morphology described in Figure 1 has been fully confirmed.

### 3.2. Mechanical Properties and Discussion

**Figure 5:**
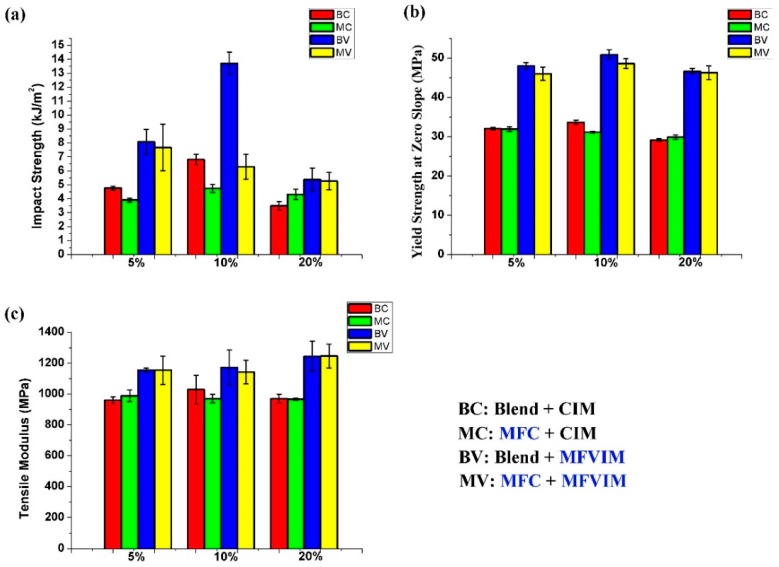
Mechanical properties of various samples. (**a**) Impact strength, (**b**) yield strength at zero slope, and (**c**) tensile modulus.

Figure 5 and Table 2 present the mechanical properties. For the BC samples (shown in red), which were prepared by blending and conventional injection molding, the 10% PET blend (BC10) obtained the highest values of impact (6.8 kJ/m^2^) and yield (33.6 MPa) strength. More PET content sharply decreased the impact strength. The impact strength of BC20 was only 3.5 kJ/m^2^, which corresponds to a decrease of approximately 50%. Some researchers believe that voids and stress concentration at high filler volume percentages are the main reasons for composite strength reduction [40,41]. As shown in Figure 5a, when PET fiber content was low, the PET fiber could decrease the impact strength, so the impact strengths of MC5 and MC10 were lower than that of BC5 and BC10, respectively. However, the impact strength of MC20 was 23% higher than that of BC20. This means that higher PET fiber content can increase impact strength. Meanwhile, at higher PET fiber content, the yield strength slightly increases, and the tensile modulus remains constant.

According to the comparison of BC (red) and BV (blue) in Figure 5, changing the PP matrix crystal structure from a spherulite to a shish-kebab can effectively increase the mechanical properties. For the 5% PET blend, the impact strength, yield strength, and tensile modulus of BV5 relative to BC5 increased by 60%, 50%, and 20%, respectively. Moreover, the performance improvement values were 101%, 51%, and 14%; and 54%, 62%, and 28% for the 10% and 20% PET, respectively. Among these samples, BV10 obtained the highest impact (13.7 kJ/m^2^) and yield (50.9 MPa) strength. More PET content (BV20) increased the tensile modulus but decreased the impact and yield strength.

However, MFC and MFVIM technologies should not be used simultaneously. As shown in Figure 5, the performance of MV (yellow) was always lower than that of BV (blue). This condition is due to the PET microfibers that hinder the flow of the PP melt, thereby changing the direction and strength of the imposed shear stress during injection molding. This approach decreases the intensity of the shish-kebab. This phenomenon also occurred in our previous experiments when glass fibers were added to PP [42]. Moreover, the high shear stress necessary to form a shish-kebab can break and decrease the performance of PET fibers.

**Figure 6:**
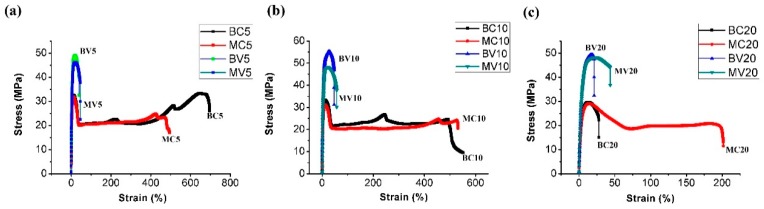
Selected stress–strain curves of various samples with different PET contents: (**a**) 5%, (**b**) 10% and (**c**) 20%.

Figure 6 illustrates the stress–strain behavior of various samples. In summary, the elongation at the break of BV and MV was considerably lower than that for BC and MC. These results indicate that shish-kebabs can increase tensile and impact strength but decrease ductility because the PP chains in shish-kebabs are already oriented and cannot be further stretched. When the PET content reaches 20%, the elongation at the break evidently decreases, especially for BC20. The decreased ductility is due to the constant percentage of POE-*g*-MA. Therefore, the increased PET content can thin the POE-*g*-MA coating layer. Thus, the stress transfer between the PP matrix and the PET fiber is limited and ductility is decreased. As shown in Figure 6c, the MFC technology can effectively improve the elongation at the break of MC20, relative to BC20, to approximately 200%. In this case, the stress transfer between the PP matrix and the PET fiber was enhanced; that is, the PET fibers do most of the load bearing until they finally fail together. 

### 3.3. Thermal Behaviour

**Figure 7:**
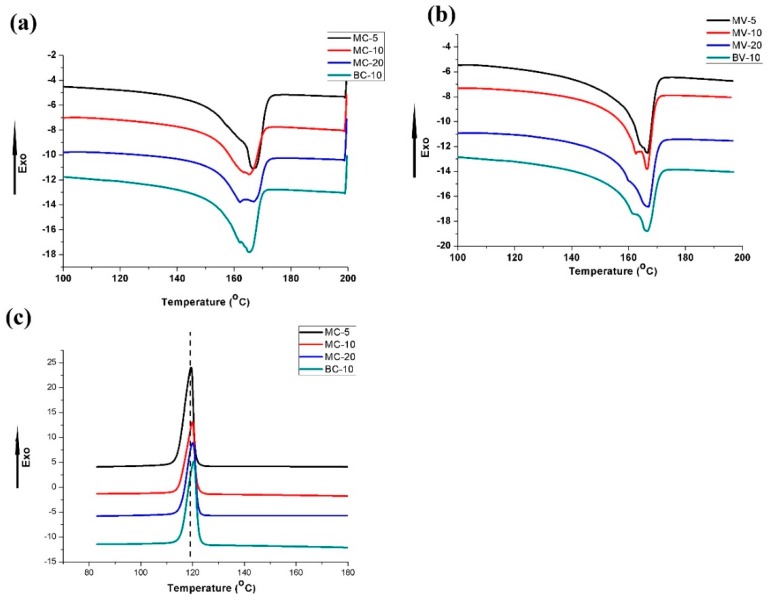
Differential scanning calorimetry (DSC) curves: heating processes of samples prepared by (**a**) CIM and (**b**) MFVIM; (**c**) cooling process.

The thermal behavior of all samples was evaluated by DSC experiments. The heating and cooling curves are illustrated in Figure 7, and the related values are listed in Table 3. The PP melting peaks were observed at approximately 166 °C for all samples. Moreover, remarkable shoulder peaks were obtained at approximately 162 °C for MC20, MV10, and BV10. Other samples also tended to form shoulder peaks that are not evident. The formation of a shoulder peak may be related to POE-*g*-MA because the POE backbone is mostly amorphous and only a small amount of crystallization can occur at low temperatures. Thus, POE-*g*-MA remains in the molten state during the crystallization of PP. The PP molecular chains can penetrate into the POE phase due to a certain degree of miscibility of PP and POE [43,44]. Finally, this condition may affect the crystallization of PP and induce the formation of a shoulder peak. As shown in Figure 7c, the crystallization temperature (T_c_) slightly increased with a rise in PET content. This condition indicates that PET will slightly promote the crystallization of PP, and that the nucleation ability of spherical PET particles is greater than that of PET fibers. As found in previous work, PET can considerably promote the crystallization of PP, but POE-*g*-MA will cover PET, thereby inhibiting the nucleating effect of PET for the PP matrix [21]. Besides this, due to the nucleating ability of PET, the highest PET content always lead to the highest crystallinity (*X_c_*), as shown in Table 3. 

### 3.4. Fracture Mechanism

**Figure 8:**
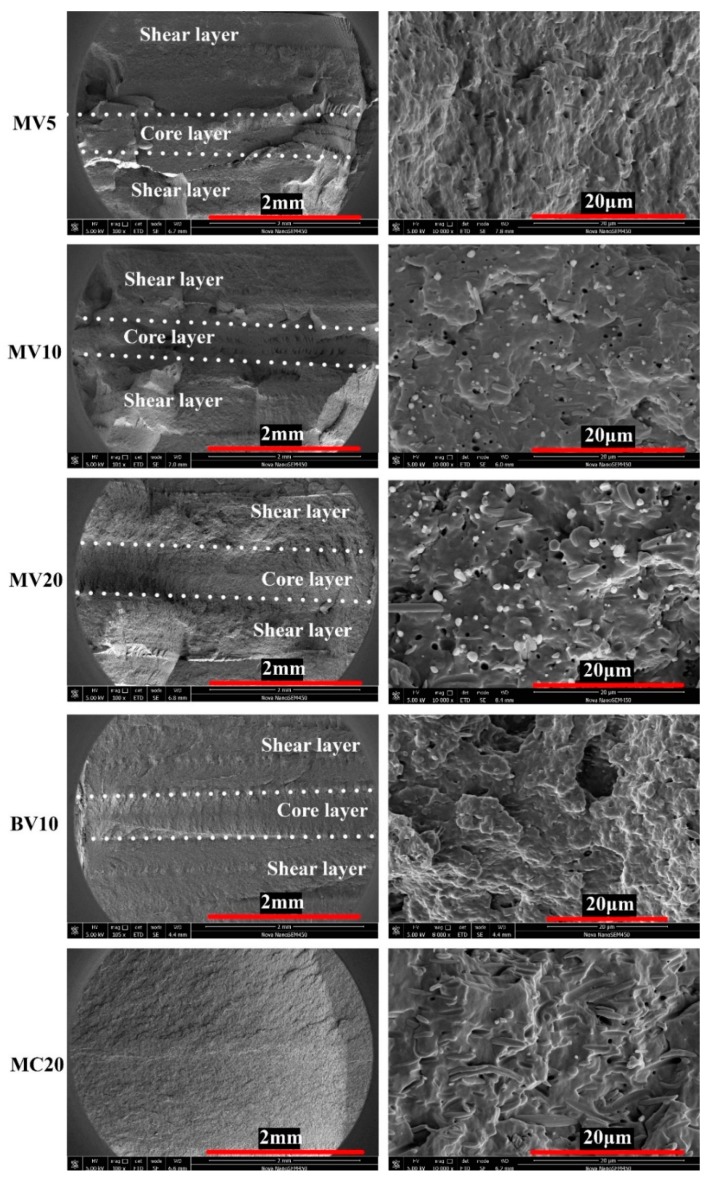
SEM of impact fracture surfaces of selected samples.

The fracture surfaces of the impacted samples were investigated by using SEM to further study the fracture mechanism. The corresponding images are shown in Figure 8. The overview is shown in the left column of the figure. For MV5, MV10, MV20, and BV10, the fracture surface can be divided into shear and core layers by changing the morphology due to the different fracture mechanisms of spherulites and shish-kebabs [34]. As described previously, the impact strength of MV5 is better than that of MV10 and MV20. This difference is due to the PET microfibers that block the flash groove and prevent the occurrence of a multiflow, thereby decreasing the shish-kebab content. Accordingly, the thickness of the shear layer of MV20 is lower than that of MV5 and MV10. The shear layer of MC20 is too thin to cause a separated fracture morphology, and its fracture surface is smoother than that of the other samples. Therefore, MC20 obtained the lowest impact strength among all samples (Figure 8).

The right column in Figure 8 is a partial enlargement view of the shear layer (MC20 is the core layer). Finding tiny microfibers for MV5 was difficult, while considerable holes and fibers were observed for MV20. In MV20, the fibers are perpendicular to the viewing surface. Thus, these fibers resemble spots. By contrast, the fibers in MC20 are randomly oriented. The detailed fracture surface of BV10 is rougher and coarser than those of the other samples and is in accordance with its highest impact strength.

### 3.5. Core–Shell Structure

**Figure 9:**
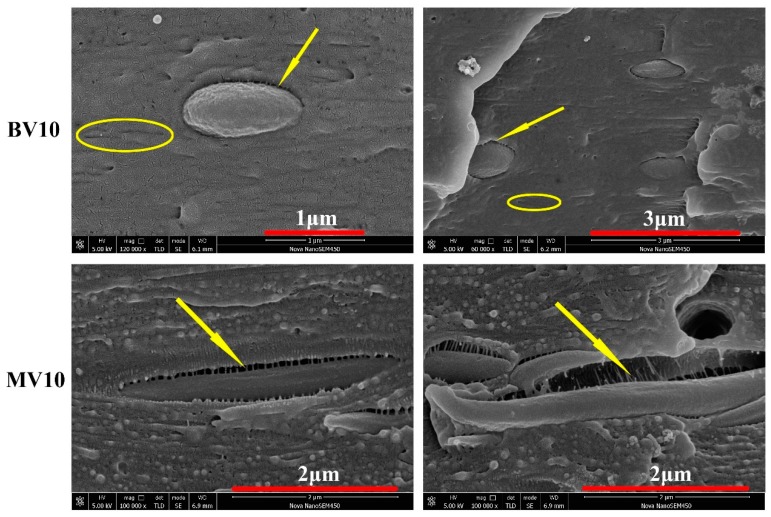
Core–shell structures. Arrows indicate POE-*g*-MA removed by n-heptane.

Further studying the distribution of POE-*g*-MA can help us to understand the manner by which BV10 obtains the highest impact and yield strength. Some evident gaps can be observed between PET and the PP matrix in Figure 9, and they indicate that the POE-*g*-MA phase was removed by n-heptane. Moreover, core–shell particles were formed in this work. Some long grooves (marked by circles) can also be observed, and they indicate that some POE-*g*-MA phases were dispersed in the matrix and failed to attach to PET. Evidently, for core–shell structures, POE-*g*-MA acts as the shell, and PET forms the core. Previous research proved that the toughening effect of elongated elastomer and spherical particles is not oriented along the flow direction due to the poor ability to initialize massive crazing and micro voiding [45,46,47]. Therefore, the impact strength of BV10 is higher than that of MV10. For BV10, an intense shear yielding phenomenon also occurs owing to the spherical core–shell structure. Cavitation could occur at the internal and external parts of the core–shell particles that can absorb energy [48]. BV10 obtained the highest impact and yield strength because of the combination of the fine core–shell structure and high shish-kebab content.

## 4. Conclusions

This work presented a comprehensive study on the relationship between the microstructure and the mechanical properties of PP/PET. Four types of PP/PET blends were prepared by different extrusion and injection methods. Different types of processing methods could be chosen to obtain the best results with changing PET content. When the PET content is low (5%/10%), the MFC technology cannot form sufficient microfibers to improve the mechanical properties. When the PET content is high and reaches 20%, the MFC technology can play an enhanced role (especially in terms of impact and strain at break). By contrast, MFVIM considerably improves the properties at low PET contents. Specifically, when the PET content is 10%, the core–shell PET/POE-*g*-MA spherules and shish-kebabs improve the yield and impact strength. The PET fiber and shish-kebab should not exist simultaneously because PET fibers hinder the flow of PP melt, thereby decreasing the intensity of shish-kebabs. Moreover, the high shear stress that is necessary to form a shish-kebab can break the PET fiber. Consequently, the performance of the PET fiber is diminished. 

## Figures and Tables

**Table 1:** Blend composition and preparation method.

Sample	Extrusion Method	Injection Method	PET (wt)%	POE-*g*-Ma (wt)%
BC 5/10/20	Simple Blend	CIM	5/10/20	4
MC 5/10/20	MFC	CIM	5/10/20	4
BV 5/10/20	Simple Blend	MFVIM	5/10/20	4
MV 5/10/20	MFC	MFVIM	5/10/20	4

**Table 2:** Mechanical properties.

Samples	BC5	BC10	BC20	MC5	MC10	MC20	BV5	BV10	BV20	MV5	MV10	MV20
**Yield Strength (MPa)**	32.0 ± 0.3	33.6 ± 0.6	29.1 ± 0.4	31.2 ± 0.6	31.1 ± 0.2	29.9 ± 0.6	48.0 ± 0.8	50.9 ± 1.2	46.6 ± 0.7	46.0 ± 1.7	48.6 ± 1.2	46.3 ± 1.8
**Tensile Modulus (MPa)**	959 ± 21	1029 ± 92	970 ± 28	989 ± 37	970 ± 27	966 ± 7	1155 ± 13	1172 ± 113	1245 ± 98	1154 ± 92	1142 ± 77	1246 ± 77
**Impact Strength (kJ/m^2^)**	4.7 ± 0.1	6.8 ± 0.4	3.5 ± 0.3	3.9 ± 0.1	4.8 ± 0.3	4.3 ± 0.4	8.1 ± 0.9	13.7 ± 0.8	5.4 ± 0.8	7.7 ± 1.7	6.3 ± 0.9	5.3 ± 0.6

**Table 3:** Thermal properties during heating and cooling.

Samples	T_m_ (°C)	T_c_ (°C)	Δ*H* (J·g^−1^)	*X_c_* (%)
**MC5**	166.8	119.4	70.78	36
**MC10**	165.3	119.9	64.44	35
**MC20**	167.0/161.9	120.0	62.03	37
**BC10**	165.1	120.4	66.23	36
**MV5**	166.6	\	70.93	36
**MV10**	166.4/162.5	\	68.38	37
**MV20**	167.0	\	60.89	37
**BV10**	166.4/161.3	\	67.48	36

## Supplementary Materials

The following are available online at http://www.mdpi.com/2073-4360/11/2/248/s1, Figure S1: Schematic of multiflow vibrate-injection molding: (a,b) first flow; (c,d) second flow; and (e,f) third flow.
