# Supplementary material for: Effects of Phase Morphology on Mechanical Properties: Oriented/Unoriented PP Crystal Combination with Spherical/Microfibrillar PET Phase
Source: Polymers (Basel). 2019 Feb 2;11(2):248. doi: 10.3390/polym11020248 (PMC6419086; doi:10.3390/polym11020248)
Supplement: Supplementary file 1 [file polymers-11-00248-s001.pdf]

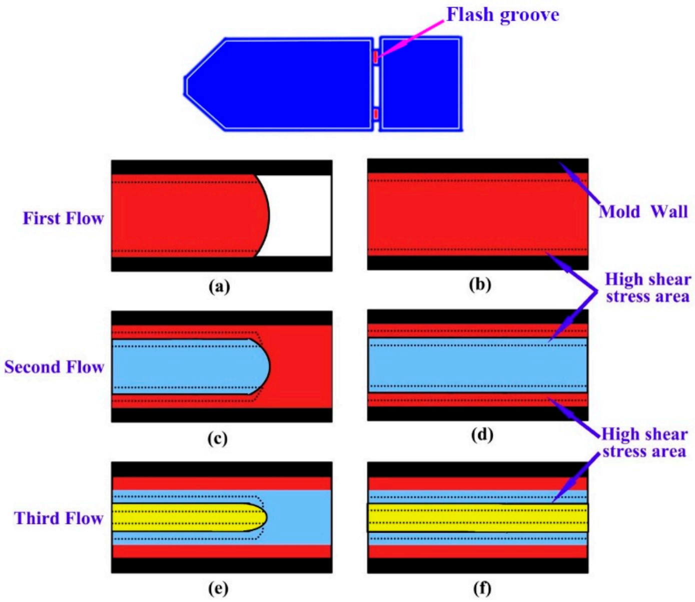

Figure S1. Schematic of multiflow vibrate-injection molding: (a, b) first flow; (c, d) second flow; and (e, f) third flow.

As shown in Figure S1, the mold is initially filled with melt at a certain injection pressure, and no melt could spill through the flash groove. The oscillatory pressure is subsequently introduced to form a second/third flow during the packing stage, and a part of the melt could be pushed out of the cavity through the flash groove.
